# Assessment of depressive symptoms in patients with COVID-19 during the second wave of epidemic in Myanmar: A cross-sectional single-center study

**DOI:** 10.1371/journal.pone.0252189

**Published:** 2021-06-04

**Authors:** Ye Minn Htun, Kyaw Thiha, Aung Aung, Nay Myo Aung, Thet Wai Oo, Pyae Sone Win, Nay Hein Sint, Kaung Myat Naing, Aung Khant Min, Kyaw Myo Tun, Kyaw Hlaing

**Affiliations:** 1 Department of Prevention and Research Development of Hepatitis, AIDS and Other Viral Diseases, Health and Disease Control Unit, Nay Pyi Taw, Myanmar; 2 Department of Mental Health, Defence Services Medical Academy, Yangon, Myanmar; 3 Department of Research and Development, Defence Services Medical School, Yangon, Myanmar; 4 Department of Chest Medicine, Defence Services Medical Academy, Yangon, Myanmar; 5 Special Operation Medical Research Department, Defence Services Medical Research Centre, Nay Pyi Taw, Myanmar; 6 Department of Preventive and Social Medicine, Defence Services Medical Academy, Yangon, Myanmar; All India Institute of Medical Sciences Jodhpur, INDIA

## Abstract

**Background:**

Coronavirus disease 2019 (COVID-19) pandemic has had a great impact on every aspect of society. All countries launched preventive measures such as quarantine, lockdown, and physical distancing to control the disease spread. These restrictions might effect on daily life and mental health. This study aimed to assess the prevalence and associated factors of depressive symptoms in patients with COVID-19 at the Treatment Center.

**Methods:**

A cross-sectional telephone survey was carried out at Hmawbi COVID-19 Treatment Center, Myanmar from December 2020 to January 2021. A total of 142 patients with COVID-19 who met the criteria were invited to participate in the study. A pre-tested Center for Epidemiologic Studies Depression Scale (CES-D) was used as a tool for depressive symptoms assessment. Data were analyzed by using binary logistic regression to identify associated factors of depressive symptoms. Adjusted odds ratio (AOR) with a 95% confidence interval (CI) was computed to determine the level of significance with a p < 0.05.

**Results:**

The prevalence of depressive symptoms in patients with COVID-19 was 38.7%, with the means (± standard deviation, SD) subscale of somatic symptom, negative effect, and anhedonia were 4.64 (±2.53), 2.51 (± 2.12), and 5.01 (± 3.26), respectively. The patients with 40 years and older (AOR: 2.99, 95% CI: 1.36–6.59), < 4 of household size (AOR: 3.45, 95% CI: 1.46–8.15), ≤ 400,000 kyats of monthly family income (AOR: 2.38, 95% CI: 1.02–5.54) and infection to family members (AOR: 4.18, 95% CI: 1.74–10.07) were significant associated factors of depressive symptoms.

**Conclusion:**

The high prevalence of depressive symptoms, approximately 40%, was found in patients with COVID-19 in the Treatment Center. Establishments of psychosocial supports, providing psychoeducation, enhancing the social contact with family and friends, and using credible source of information related COVID-19 would be integral parts of mental health services in COVID-19 pandemic situation.

## Introduction

In December 2019, China reported an outbreak of coronavirus disease 2019 (COVID-19) caused by severe acute respiratory syndrome coronavirus 2 (SARS–CoV2) in Wuhan, the capital city of Hubei province [1–3]. The virus can widely spread and become a great global public health concern. On 11^th^ March 2020, the World Health Organization (WHO) declared the COVID-19 as a pandemic [4], and then the Americas and Europe regions were more affected than other regions [5]. As of 7^th^ March 2021, the total number of cases of infection was 116 million around the world with approximately 2.5 million deaths. In Myanmar, the first cases of COVID-19 were reported on 23^rd^ March 2020 and currently, all regions and states were affected. On 7^th^ March 2021, there were a total of 142,023 COVID-19 confirmed cases and 3,200 deaths in Myanmar [6].

As a response to prevent the spread of COVID-19, many countries were using a combination of containment and mitigation activities, while focusing on the most vulnerable people including elderly people and those with comorbidities [7]. The governments have enforced restrictions on national and international travel, border closures, and quarantine of suspected people [8]. Moreover, the community mitigation strategies or non-pharmaceutical interventions (such as hand washing, wearing the mask, and keeping a distance from sick people), social distancing policies (school closure, work from home, stay at home except for essential workers, and avoidance of the public gathering), quarantine the close contacts, expansion of diagnostic testing, screening for the high-risk persons, contact tracing and use of surveillance applications were implemented [7, 9].

The rapid transmission of the virus around the world caused global economic and social disruptions. The COVID-19 pandemic has cruelly affected the healthcare, education, food, and financial systems of all countries. It also created noteworthy knock-on effects on both the daily life of peoples and the global economy including production, investment, and employment. Consequently, most of the countries have slowed down their manufacturing of the products [10, 11]. Due to extensive business closures, especially in lower-income populations, the national economic crisis was expected to lead to a dramatic rise in unemployment and mental health problems [12]. With little or no income during lockdowns, many people with informal economic were unable to feed themselves and their families. Therefore, informal workers were particularly vulnerable due to a lack of access to quality health care and social protection [13].

During the COVID-19 pandemic, facing with new realities such as working from home, temporary unemployment, lack of physical contact with other family members, friends, and colleagues, seeing or being aware of critically ill beloved one affected by the coronavirus, the passing of beloved one or even thinking of their health could create the development of anxiety, panic attacks, depression, and other mental illnesses [14]. The worry about financial situation or job, or loss of support services, changes in sleep or eating patterns, difficulty concentrating, and worsening of chronic diseases could induce deteriorating of mental health conditions [15]. The major stressors such as uncertainty of prognosis, severe shortages of resources for testing and treatment, the imposition of unfamiliar public health measures that infringe on personal freedoms, financial losses, and inconsistent information from authorities would contribute to widespread emotional distress [16].

Moreover, the patients after diagnosis of COVID-19 were more likely to have psychological concerns such as anxiety for unpredictability of disease, fear of progression of their illness, disability, or premature death [17, 18]. Early identification of individuals in the initial stages of a psychological disorder could make effective intervention strategies [19]. Detection of the levels of distress and other factors associated with a higher likelihood of developing psychological problems are crucial for providing behavior guidance and psychological support for the patients with COVID-19. In Myanmar, the prevalence of depression was 6 per 100,000 population in 2016 [20]. Mental health services were not prioritization in primary health care due to there was no medical policy issued regarding mental health. Lack of evidence on prevalence and geographic spread were major barriers to tackling the problems of mental health [21]. In addition, there was limited information related to the risk factors for depressive symptoms in patients with COVID-19. Therefore, this study was designed to assess the prevalence of depressive symptoms and to find out its related factors among patients with COVID-19 in Treatment Center.

## Materials and methods

### Study design and setting

A cross-sectional study was conducted at Hmawbi Treatment Centre, Yangon Region, Myanmar from December 2020 to January 2021. There were twelve treatment centers for COVID-19 in Yangon Region and the patients with serious symptoms are being mainly treated at designated hospitals such as Waibargi, South Okkalapa, and Phaunggyi hospitals. Hmawbi Treatment Centre was established in the second wave of the COVID-19 epidemic. It was located in the Northern District of Yangon Region and approximately 50 km far from Yangon City.

### Study population

The study population was patients with COVID-19 attending in Hmawbi Treatment Center during the study period.

### Sample size determination and sampling technique

The sample size was calculated using single proportion formula with an assumption of 95% Confident Interval (CI), 8% margin of error, and 31.2% of depressive symptom in SARS patients [22]. Therefore, the total sample size was determined as 142 after adding 10% of the non-response rate. The Hmawbi Treatment Centre was purposively selected due to one of the isolation hospitals which provided the medical treatment to COVID-19 confirmed cases who were different background and settings. All registered patients with confirmed SARS-CoV-2 infection by positive results on reverse transcription polymerase chain reaction testing of a nasopharyngeal sample, who consented to the study, and who were older than 18 years were eligible to participate in the study. The patients who were being treated with high flow O_2_ 5L/min, fluid therapy, IV drugs, ventilators and specialist assess needed, and referred to intensive care unit were excluded.

### Data collection procedure and tools

Considering the transmission and severity of the COVID-19 epidemic, the data were collected through the telephone interview using pretested structured questionnaire instead of a face-to-face interview after getting verbal informed consent. The background characteristics and telephone numbers of patients were documented in patient records that were kept in the administrative office. The three interviewers explained the purposes of the study before starting the telephone interview. The questionnaire comprised two parts: background characteristics (including socioeconomic factors, epidemic-related factors, psychological factors, and psychosocial supports) and assessment for depressive symptoms.

The Center for Epidemiologic Studies Depression (CES-D) scale was used for the assessment of depressive symptoms in patients with COVID-19. The CES-D scale which was originally published by Radloff in 1977, was a short assessment tool designed to measure the current level of depressive symptoms in the clinical setting and general population [23]. It contained 20 items about symptoms that occurred in a week prior to the interview with response options from 0 to 3 that refer to the frequency of the symptoms (0 = rarely or none of the time, 1 = some or a little of the time, 2 = occasionally or a moderate amount of the time, 3 = most or all of the time). The scoring of positive items (4, 8, 12, and 16) was reversed. The total scores range from 0 to 60, with high scores indicating greater depressive symptoms. The recommended cutoff point for depressive symptoms was 16 that assisted in identifying individuals at risk for clinical depression, with good sensitivity, specificity, and high internal consistency [24–26]. In the CES-D scale, there were three subscales or factor structures that included somatic symptoms (items 1, 2, 5, 7, 11, 20), negative affect (items 3, 6, 14, 18), and anhedonia (items 4, 8, 12, 16) [26].

The questionnaire was originally developed in English and then translated to Myanmar (local language). It was pre-tested in a Treatment Centre which was similar settings to those of the study population and refined to more improvement of validity and reliability which was confirmed by Cronbach’s α of 0.89.

### Operational definitions

The living situation was described as living with whom the patients live (such as alone, with family members and with friends). Household size was defined as the total number of people living in a home and categorized as < 4 and ≥ 4 household members according to the Myanmar Living Conditions Survey, 2017 that estimated an average of 4 people living in each household [27]. Monthly family income was categorized into two groups: ≤ 400,000 and > 400,000 kyats. An average of monthly family income, 400,000 kyats, was used as a cutoff value according to the Myanmar Data Collection Survey on Housing Finance System Report, 2018 [28]. The smoking status was defined as the patient who has smoked 100 cigarettes in his or her lifetime and those who currently smoke cigarettes or cheroots. Alcohol drinking was defined as the patient who takes more than three drinks per week on average over the past year. The comorbidity was the presence of one and more diseases which was chronic or long-term in the same patients. According to the case definition of the Ministry of Health and Sports, Myanmar, the contact with known COVID-19 case was defined as a person who experienced any one of the following exposures during the 2 days before and the 14 days after the onset of symptoms of a probable or confirmed case, who are face-to-face contact with a probable or confirmed case within 1 meter and for more than 15 minutes, direct physical contact with a probable or confirmed case, direct care for a patient with probable or confirmed COVID-19 disease without using proper personal protective equipment, or other situations as indicated by local risk assessments [29].

The travelling history abroad was a patient with COVID-19 who had a history of travelling and come back from foreign countries within the past 14 days [29, 30]. Travelling history to townships under stay at home order was described as a patient with COVID-19 who had a history of travel to the township under stay at home order within the past 14 days. Presenting symptom was a symptom or problem that was offered by the patient with COVID-19 as a reason for seeking treatment. Social support was defined as providing the support of direct material aid, advice, suggestions, the existence of social networks, community relations, understanding of emotional experience, and satisfaction. Mental support was defined as psychological assistance services including telephone, internet, and application-based counseling or intervention. As the outcome variable, depressive symptoms were the combined signs, markers, and indications of depression that is a mental disorder that presents with depressed mood, loss of interest or pleasure, decreased energy, feelings of guilty and poor concentration [31].

### Data quality control

To ensure consistency of the data collection process, the principal investigator provided one-day intensive training to another two data collectors regarding the explanation of study objectives, the data collection tools, informed consent, and procedures. Daily supervision of data collection was made at the control room of the Treatment Center during the study period by the principal investigator. Then, the principal investigator immediately reviewed the collected data for completeness and consistency.

### Statistical analysis

After coding, the data entry was done by using Microsoft Excel 2016. The data were cleaned for consistency and then exported to IBM SPSS Statistics for Windows, Version 23.0 (Armonk, NY: IBM Corp) for further analysis. Descriptive statistics were presented as frequency and percentages for categorical variables and mean and standard deviation (SD) for continuous variables. Both bivariable and multivariable logistic regression models were used in the data analysis. The variables with p value < 0.05 in the bivariable logistic regression analysis were retained in the backward stepwise multivariable logistic regression analysis to determine the independent predictors of depressive symptoms in patients with COVID-19. Age, household size, monthly family income, smoking status, comorbidity, infection to family members, and mental supports were included in the multivariable logistic regression model. Both crude odds ratio (COR) and adjusted odds ratio (AOR) with a 95% confidence interval (CI) were estimated to show the strength of association. The independent variables associated with the dependent variable were declared when AOR with 95% CI was significant in the multivariable analysis at p < 0.05.

### Ethical considerations

This study was reviewed and approved by the Institutional Review Board (IRB) of Defence Services Medical Research Centre, Nay Pyi Taw (IRB/2020/A-05). The permission for data collection was obtained from an authorized person of Hmawbi Treatment Center. Then, verbal informed consent approved by the IRB was obtained by using a telephone from each participant before starting the interview. For the agreement of informed consent, the voice recording by telephone was done to minimize the risk of disease spread. All participants have explained the objectives, risks, and benefits related to the study. Moreover, all participants were informed about the right of withdrawing from the study without restriction whenever necessary. Privacy and confidentiality of all information obtained from the participants were maintained throughout the study process.

## Results

A total of 142 patients with COVID-19 participated in this study and the background characteristics of patients with COVID-19 were shown in Table 1. Of all participants, 97 (68.3%) were male and 45 (31.7%) were female. The mean (± SD) age of the patients was 38.75 (± 14.69) years with a range of 18–79 years, and 87 (61.3%) patients were younger than 40 years. Among the total, 33 (23.2%) and 28 (19.7%) patients were from Hlaing Township and Mingalardon Township respectively. Most of the patients, 93 (65.5%), had achieved high school education and below and 112 (78.9%) were patients employed. In this study, there were more married patients, 87 (61.3%), than the single, 55 (38.7%). Of all patients, 107 (75.4%) were living with their family members, 73 (51.4%) were living with less than 4 household members, and 77 (54.2%) had 400,000 kyats and less of monthly family income. Only 12 (8.5%) and 9 (6.3%) patients were smokers and alcohol drinkers, respectively, and 20 (14.1%) patients had comorbidities.

**Table 1:** Background characteristics of patients with COVID-19.

Variables		n (%)
Sex		
	Male	97 (68.3)
	Female	45 (31.7)
Age		
	< 40 years	87 (61.3)
	≥ 40 years	55 (38.7)
	Mean (± SD); 38.75 (± 14.69) years, Minimum 18, Maximum 79 years	
Township		
	Mayangone	8 (5.6)
	Hmawbi	8 (5.6)
	Insein	13 (9.2)
	Mingalardon	28 (19.7)
	Hlaing	33 (23.2)
	Others	52 (36.6)
Education		
	High school education and below	93 (65.5)
	University and graduate	49 (34.5)
Occupation		
	Dependent	30 (21.1)
	Employed	112 (78.9)
Marital status		
	Single	55 (38.7)
	Married	87 (61.3)
Living situation		
	Living alone	10 (7.0)
	Living with family members	107 (75.4)
	Living with friends	25 (17.6)
Household size		
	< 4	73 (51.4)
	≥ 4	69 (48.6)
	Mean (± SD); 3.66 (± 1.82), Minimum 1, Maximum 10	
Monthly family income		
	≤ 400,000 kyats	77 (54.2)
	> 400,000 kyats	65 (45.8)
	Mean (± SD); 466,373.24 (± 321,212.78) kyats, Minimum 180,000, Maximum 3,000,000 kyats	
Smoking status		
	Yes	12 (8.5)
	No	130 (91.5)
Alcohol drinking		
	Yes	9 (6.3)
	No	133 (93.7)
Comorbidity		
	Yes	20 (14.1)
	No	122 (85.9)

As shown in Table 2, 78 (54.9%) patients had a history of contact with COVID-19 confirmed cases and 42 (29.6%) gave a history of infection in their family members. Only two (1.4%) patients travelled abroad last 14 days and 27 (19.0%) travelled to townships under stay at home restriction. Among total patients, 96 (67.6%) were symptomatic patients. In psychological factors, two (1.4%) patients had psychological history and five (3.5%) had a psychological history in their family members. With regard to psychosocial supports received in the course of treatment, 91 (64.1%) patients received social supports and 101 (71.1%) got mental supports.

**Table 2:** Epidemic-related factors, psychological factors, and psychosocial supports of patients with COVID-19.

Variables			n (%)
**Epidemic-related factors**			
	Contact history		
		Yes	78 (54.9)
		No	64 (45.1)
	Infection to family members		
		Yes	42 (29.6)
		No	100 (70.4)
	Travel history abroad		
		Yes	2 (1.4)
		No	140 (98.6)
	Travel history to townships under stay at home restriction		
		Yes	27 (19.0)
		No	115 (81.0)
	Presenting Symptom		
		Symptomatic	96 (67.6)
		Asymptomatic	46 (32.4)
**Psychological factors**			
	Psychological history		
		Yes	2 (1.4)
		No	140 (98.6)
	Psychological history in family members		
		Yes	5 (3.5)
		No	137 (96.5)
**Psychosocial supports**			
	Social support		
		Yes	91 (64.1)
		No	51 (35.9)
	Mental support		
		Yes	101 (71.1)
		No	41 (28.9)

The depressive symptoms of patients with COVID-19 were presented in Table 3. The total mean (± SD) score of CES-D was 16.04 (± 7.15) and 55 (38.7%) patients had depressive symptoms. In subscales of depressive symptoms, the mean (± SD) scores for the somatic symptom, negative effect, and anhedonia were 4.64 (± 2.53), 2.51 (± 2.12), and 5.01 (± 3.26), respectively.

**Table 3:** Depressive symptoms in patients with COVID-19.

Variables			Value
CES-D score, mean (± SD)			
	Total score		16.04 (± 7.15)
		Somatic symptom subscale	4.64 (± 2.53)
		Negative effect subscale	2.51 (± 2.12)
		Anhedonia subscale	5.01 (± 3.26)
Depressive symptoms, n (%)			
	No (< 16 CES-D score)		87 (61.3)
	Yes (≥ 16 CES-D score)		55 (38.7)

The associated factors of depressive symptoms were presented in Table 4. On bivariable logistic regression analysis, aged 40 and older (COR: 2.98, 95% CI: 1.47–6.02), less than 4 persons of household size (COR: 3.32, 95% CI: 1.63–6.79), 400,000 kyats and less of monthly family income (COR: 2.75, 95% CI: 1.35–5.60), smoking (COR: 3.53, 95% CI: 1.01–12.36), comorbidity (COR: 2.76: 95% CI: 1.05–7.26), infection to family members (COR: 2.97, 95% CI: 1.41–6.24), and mental support (COR: 2.39, 95% CI: 1.14–5.01) were significantly associated factors of depressive symptoms. After adjusting to confounding factors, aged 40 and older (AOR: 2.99, 95% CI: 1.36–6.59), less than 4 persons of household size (AOR: 3.45, 95% CI: 1.46–8.15), 400,000 kyats and less of monthly family income (AOR: 2.38, 95% CI: 1.02–5.54), and infection to family members (AOR: 4.18, 95% CI: 1.74–10.07) were remained as significant associated factors of depressive symptoms.

**Table 4:** Bivariable and multivariable logistic regression analysis of factors associated with depressive symptoms in patients with COVID-19.

Variables			Depressive symptoms		p value	COR (95% CI)	p value	AOR (95% CI) ^†^
			No n (%)	Yes n (%)				
**Socioeconomic factors**								
	Sex							
		Female	31 (68.9)	14 (31.1)		1		
		Male	56 (57.7)	41 (42.3)	0.21	1.62 (0.77–3.43)		
	Age							
		< 40 years	62 (71.3)	25 (28.7)		1		1
		≥ 40 years	25 (45.5)	30 (54.5)	<0.01	2.98 (1.47–6.02)	<0.01	2.99 (1.36–6.59)
	Education							
		University and graduate	32 (65.3)	17 (34.7)		1		
		High school education and below	55 (59.1)	38 (40.9)	0.47	1.30 (0.63–2.67)		
	Occupation							
		Dependent	21 (70.0)	9 (30.0)		1		
		Employed	66 (58.9)	46 (41.1)	0.27	1.63 (0.68–3.87)		
	Household size							
		≥ 4	52 (75.4)	17 (24.6)		1		1
		< 4	35 (47.9)	38 (52.1)	<0.01	3.32 (1.63–6.79)	<0.01	3.45 (1.46–8.15)
	Monthly family income							
		> 400,000 kyats	48 (73.8)	17 (26.2)		1		1
		≤ 400,000 kyats	39 (50.6)	38 (49.4)	<0.01	2.75 (1.35–5.60)	0.04	2.38 (1.02–5.54)
	Smoking							
		No	83 (63.8)	47 (36.2)		1		
		Yes	4 (33.3)	8 (66.7)	0.04	3.53 (1.01–12.36)		
	Alcohol							
		No	83 (62.4)	50 (37.6)		1		
		Yes	4 (44.4)	5 (55.6)	0.29	2.08 (0.53–8.09)		
	Comorbidity							
		No	79 (64.8)	43 (35.2)		1		
		Yes	8 (40.0)	12 (60.0)	0.04	2.76 (1.05–7.26)		
**Epidemic-related factors**								
	Contact history							
		No	41 (64.1)	23 (35.9)		1		
		Yes	46 (59.0)	32 (41.0)	0.54	1.24 (0.63–2.45)		
	Infection to family members							
		No	69 (69.0)	31 (31.0)		1		1
		Yes	18 (42.9)	24 (57.1)	<0.01	2.97 (1.41–6.24)	<0.01	4.18 (1.74–10.07)
	Presenting symptom							
		Asymptomatic	33 (71.7)	13 (28.3)		1		
		Symptomatic	54 (56.3)	42 (43.8)	0.08	1.97 (0.93–4.21)		
**Psychological factors**								
	Psychological history							
		No	87 (62.1)	53 (37.9)				
		Yes	0 (0.0)	2 (100.0)				
	Psychological history in family members							
		No	84 (61.3)	53 (38.7)		1		
		Yes	3 (60.0)	2 (40.0)	0.95	1.06 (0.17–6.53)		
**Psychosocial supports**								
	Social support							
		Yes	60 (65.9)	31 (34.1)		1		
		No	27 (52.9)	24 (47.1)	0.13	1.72 (0.85–3.47)		
	Mental support							
		Yes	68 (67.3)	33 (32.7)		1		
		No	19 (46.3)	22 (53.7)	0.02	2.39 (1.14–5.01)		

COR: crude odds ratio, AOR: adjusted odds ratio.

^**†**^ All variables with p < 0.05 in bivariable analysis were included in multivariable regression model.

## Discussion

This study aimed to assess the depressive symptoms in patients with COVID-19 and to find out the associated factors with it. In sex distribution, men were more infected than women and it was in line with the findings of previous studies done in China [32, 33], and Israel [34]. However, this finding was contrary to the results of the other studies conducted in the China [19, 35] and Ecuador [36] stated that the sex distribution of patients with COVID-19 was not too different. The mean age of the patients (38.7 years) was comparable with the earlier study done in Jordan (35.8 years) and lower than the studies done in China (46.9 years) [19], (53.6 years) [37], and (50.4 years) [35] respectively, and Israel (49.3 years) [34]. A possible explanation for these results might be the differences in sociodemographic background of study areas and variation in the sample size.

The people living within a household could infect each other when one was infected. Consequently, it could impact economic deterioration or household income as a result of isolation and temporary unemployment of infected household members. In the current study, the prevalence of the infection to family members (29.6%) was lower than the findings of former studies conducted in China reported that the family members with confirmed COVID-19 were (32.7–51.2%) [17, 35, 37, 38]. It has been suggested due to disparity of adherence to preventive measure among working age population who might have greater exposure to coronavirus in daily life and workplace, and while using public transport.

The prevalence of depressive symptoms in patients with COVID-19 was (38.7%) in this study and it was relatively higher than that of studies conducted among patients with COVID-19 in China stated that the patients with depressive symptoms were (13.4–29.3%) [17, 19, 33, 37, 39]. Likewise, this result was higher than the prevalence of some published studies done in Ecuador (20.3%) [36], and Italy (31.0%) [40]. Conversely, it was lower than the findings of the previous researches done in China (43.1%) [35], Jordan (44.0%) [41], and Iran (85.8%) [42]. This inconsistency might be because of the differences in utilization of tools applied for the assessment of depressive symptoms, determining the cutoff value in the scoring system, background characteristics of patients, hospital admission criteria, and providing the appropriate information on time by the health authorities. Effective preventive measures by the governments, trust in official information, cultural factors and recent political situation were also contributed to the differences of mental health problems related COVID-19 [18].

In the current study, age was a significant predictor of depressive symptoms in patients with COVID-19. The patients aged 40 years and older were more likely to develop depressive symptom than the younger patients. The possible explanation of this finding could be due to the facts that the older aged people might concern about the financial burden, impact on family responsibility, and lack of social connectedness with family members, and helplessness in isolation period. In addition, the older patients were poor in opposition to the influence of isolation, especially limitation in social connectedness with friends, family members, and caregivers [43]. This result was in accord with earlier studies done in hospitals at Wuhan, China indicating that age was a factor affecting the rate of depressive symptoms in patients with COVID-19 [17, 33]. However, this result has not previously been described [38, 42].

The household size was also reported to be a significantly associated factor of depressive symptoms in patients with COVID-19. The odds of developing depressive symptoms among patients with less than four persons living in the same household were 3.45 times more likely to develop depressive symptoms compared with their counterparts. The possible reason might be due to lack of physical contact with and psychosocial supports from other household members, friends, and beloved ones. The COVID-19 related stressors including physical distancing, financial constraints, pre-existing health problems, stigma, and discrimination increased the chance of developing depressive symptoms. During the isolation period, social and mental supports by household members was a main role in the mental health of patients with COVID-19 [16].

The COVID-19 pandemic affected labour supply and productivity in all countries. The mitigation measures such as lockdowns, business closures, and social distancing triggered the loss of individual income with a high unemployment rate. The financial stressors related to low and regular income and temporary job loss due to COVID-19 restrictions were also potential contributors to mental health [44]. In this study, there was a significant association between monthly family income and depressive symptoms among patients with COVID-19. The patients with low family income were 2.38 times more likely to develop depressive symptoms than those with high family income. It seems possible that this result might be due to their financial burden, loss of basic amenities, and concerns about the quality of life in the future. A previous study conducted among patients with COVID-19 in China showed that high income was marginally significant associated with severe depression in patients with COVID-19 [37].

Excessive psychological pressure, guilty for transmission to their family members, and community stigma might progress the depressive symptoms in patients with infected family members [45]. In the current study, the patients with infected family members were more likely to get depressive symptoms than those with their counterparts. It could be due to the psychological burden of being a carrier, fear of transmitting the disease to others, feeling rejected and stigmatization, creating a cold atmosphere within the family, feeling uncertainty, increased risk of severity in infected family members with older aged and those with comorbid diseases. This result was in agreement with the findings of the studies done in China reported that infection to family members was a significant predictor of depressive symptoms in patients with COVID-19 [17, 35, 37–39, 46].

### Limitation

There were some limitations in this study. First and foremost, due to a cross-sectional nature of the study, it could not establish a causal relationship and the mechanism underlying the association was not directly identified. Therefore, future longitudinal studies with follow-up and intervention should be conducted to help in discovering the psychological effects of the patients. Second, this study was a single-center study and hence, further research would be needed to conduct with a large sample for the stronger association of independent predictors with depressive symptoms in patients with COVID-19. Third, comorbid diseases and the psychological history of the patients could not be assessed before the epidemic and the data were collected as self-reported. Therefore, recall bias might be present. In addition, the results might be varied to what extent the generalization to the patients with COVID-19 from other countries, although it could be consistent to those with the same geographic and the socioeconomic backgrounds. Lastly, some clinical and laboratory data determined for severity of disease that might be associated with depressive symptoms were not measured in this study.

## Conclusions

The prevalence of the depressive symptoms was approximately 40% of patients with COVID-19 in the Treatment Center. The patients with older age, less household size, low monthly family income and infection to family members were more likely to develop depressive symptoms. Psychosocial supports and psycho-educational intervention should be implemented in the Treatment Centers to reduce the depressive symptoms and other mental health disturbances related to COVID-19. Early screening for depressive symptoms, psychosocial counselling, enhancing social contact with family and friends, maintaining a healthy lifestyle such as proper diet and sleep, and using a credible source of information related to COVID-19 should be established to reduce the psychological impact of COVID-19.

## Supporting information

S1 Questionnaire English version of the questionnaire. (PDF)

S2 Questionnaire Myanmar version of the questionnaire. (PDF)

S1 Data Minimal data. (XLSX)
