# Supplementary material for: Assessment of depressive symptoms in patients with COVID-19 during the second wave of epidemic in Myanmar: A cross-sectional single-center study
Source: PLoS One. 2021 Jun 4;16(6):e0252189. doi: 10.1371/journal.pone.0252189 (PMC8177551; doi:10.1371/journal.pone.0252189)
Supplement: S1 Questionnaire — (PDF) [file pone.0252189.s001.pdf]

**QUESTIONNAIRES (English)**

**Code** ☐☐☐

| **Socioeconomic factors** | | |
|---|---|---|
| Age years | Sex | ☐ Male ☐ female |
| Township | | |
| Education<br>☐ Illiterate ☐ Middle school education level<br>☐ Read and write ☐ High school education level<br>☐ Primary school education level ☐ Graduate and above | | |
| Occupation<br>☐ Dependent<br>☐ Unskilled laborer<br>☐ Own business<br>☐ Private employee<br>☐ Government staff | | |
| Marital status<br>☐ Single<br>☐ Married<br>☐ Separate<br>☐ Divorced<br>☐ Widowed | | |
| Living situation<br>☐ Alone<br>☐ With family<br>☐ With friends<br>☐ Others, please specify …………………………………………………………………… | | |
| Household size | Monthly family income kyats | |
| Smoking status | ☐ Yes | ☐ No |
| Alcohol drinking | ☐ Yes | ☐ No |
| Comorbidity | ☐ Yes | ☐ No |

| | | |
|---|---|---|
| **Epidemic-related factors** | | |
| Contact history | ☐ Yes | ☐ No |
| Infected to family members | ☐ Yes | ☐ No |
| Travelling history to abroad | ☐ Yes | ☐ No |
| Travelling history to townships under stay at home order | ☐ Yes | ☐ No |
| Presenting symptom | ☐ Yes | ☐ No |
| **Psychological factors** | | |
| Psychological history | ☐ Yes | ☐ No |
| Psychological history in family members | ☐ Yes | ☐ No |
| **Psychosocial supports** | | |
| Social support | ☐ Yes | ☐ No |
| Mental support | ☐ Yes | ☐ No |

**Depressive symptoms in patients with COVID-19**

Below is a list of the ways you might have felt or behaved. Please tell me how often you have felt this way during the past week.

| Statements | | During the past week | | | |
|---|---|---|---|---|---|
| | | Rarely or none of the time (less than 1 day) | Some or a little of the time (1-2 days) | Occasionally or a moderate amount of time (3-4 days) | Most or all of the time (5-7 days) |
| 1 | I was bothered by things that usually don’t bother me. | ☐ | ☐ | ☐ | ☐ |
| 2 | I did not feel like eating; m appetite was poor. | ☐ | ☐ | ☐ | ☐ |
| 3 | I felt that I could not shake off the blues even with help from my family or friends. | ☐ | ☐ | ☐ | ☐ |
| 4 | I felt I was just as good as other people. | ☐ | ☐ | ☐ | ☐ |
| 5 | I had trouble keeping my mind on what I am doing. | ☐ | ☐ | ☐ | ☐ |
| 6 | I felt depressed. | ☐ | ☐ | ☐ | ☐ |
| 7 | I felt that everything I did was an effort. | ☐ | ☐ | ☐ | ☐ |
| 8 | I felt hopeful about the future. | ☐ | ☐ | ☐ | ☐ |
| 9 | I thought my life had been a failure. | ☐ | ☐ | ☐ | ☐ |
| 10 | I felt fearful. | ☐ | ☐ | ☐ | ☐ |
| 11 | My sleep was restless. | ☐ | ☐ | ☐ | ☐ |
| 12 | I was happy. | ☐ | ☐ | ☐ | ☐ |

| | | | | | |
|---|---|---|---|---|---|
| 13 | I talked less than usual. | ☐ | ☐ | ☐ | ☐ |
| 14 | I felt lonely. | ☐ | ☐ | ☐ | ☐ |
| 15 | People were unfriendly. | ☐ | ☐ | ☐ | ☐ |
| 16 | I enjoyed life. | ☐ | ☐ | ☐ | ☐ |
| 17 | I had crying spells. | ☐ | ☐ | ☐ | ☐ |
| 18 | I felt sad. | ☐ | ☐ | ☐ | ☐ |
| 19 | I felt people dislike me. | ☐ | ☐ | ☐ | ☐ |
| 20 | I could not get “going”. | ☐ | ☐ | ☐ | ☐ |
