# Supplementary material for: Assessment of depressive symptoms in patients with COVID-19 during the second wave of epidemic in Myanmar: A cross-sectional single-center study
Source: PLoS One. 2021 Jun 4;16(6):e0252189. doi: 10.1371/journal.pone.0252189 (PMC8177551; doi:10.1371/journal.pone.0252189)
Supplement: S2 Questionnaire — (PDF) [file pone.0252189.s002.pdf]

QUESTIONNAIRES (Myanmar)

ကုဒ်နံပါတ် ☐☐☐

**လူမှုစီးပွားရေးဆိုင်ရာအချက်အလက်များ**		
အသက် (ပြည့်ပြီး) နှစ်	☐ ကျား	☐ မ
မြို့နယ်		
ပညာအရည်အချင်း		
☐ စာမတတ်	☐ အလယ်တန်းပညာ	
☐ ရေး/ဖတ်	☐ အထက်တန်းပညာ	
☐ မူလတန်းပညာ	☐ ဘွဲ့ရနှင့် အထက်	
အလုပ်အကိုင်		
☐ မှီခို		
☐ ကျပန်း/နေ့စား		
☐ ကိုယ်ပိုင်		
☐ လခစား/ကုမ္ပဏီဝန်းထမ်း		
☐ အစိုးရဝန်ထမ်း		
အိမ်ထောင်ရေးအခြေအနေ		
☐ လူပျို/အပျို		
☐ အိမ်ထောင်သည်အတူနေ		
☐ အိမ်ထောင်သည်အတူမနေ		
☐ အိမ်ထောင်ကွဲ		
☐ မုဆိုးမ/ဖို		
နေထိုင်မှုအခြေအနေ		
☐ တစ်ကိုယ်တည်း		
☐ မိသားစုနှင့်အတူ		
☐ သူငယ်ချင်း/မိတ်ဆွေများနှင့်အတူ		
☐ အခြား ……………………………………………………………………………		
အတူနေလူဦးရေ	မိသားစုတစ်လဝင်ငွေ ကျပ်	
ဆေးလိပ်သောက်သုံးမှု	☐ သောက်ပါသည်	☐ မသောက်ပါ
အရက်သောက်သုံးမှု	☐ သောက်ပါသည်	☐ မသောက်ပါ
ရောဂါအခံ	☐ ရှိပါသည်	☐ မရှိပါ

**COVID-19 ကူးစက်ရောဂါနှင့်ပတ်သတ်သောအချက်အလက်များ**		
COVID-19 ရောဂါရှိသူနှင့် ထိတွေ့မှု	☐ ရှိပါသည်	☐ မရှိပါ
မိသားစုဝင်များအား ကူးစက်ခံရမှု	☐ ရှိပါသည်	☐ မရှိပါ
နိုင်ငံခြားသို့ လွန်ခဲ့သော ၁၄ ရက်အတွင်း သွားရောက်ခဲ့ခြင်း	☐ ရှိပါသည်	☐ မရှိပါ
Stay at home order တွင်အကျုံးဝင်သော မြို့နယ်များသို့ လွန်ခဲ့သော ၁၄ ရက်အတွင်း သွားရောက်ခဲ့ခြင်း	☐ ရှိပါသည်	☐ မရှိပါ
ရောဂါလက္ခဏာများ	☐ ရှိပါသည်	☐ မရှိပါ
**စိတ်ရောဂါနှင့်ပတ်သတ်သောအချက်အလက်များ**		
ကိုယ်တိုင် စိတ်ရောဂါခံစားနေရခြင်း	☐ ရှိပါသည်	☐ မရှိပါ
မိသားစုဝင်များတွင် စိတ်ရောဂါခံစားနေရခြင်း	☐ ရှိပါသည်	☐ မရှိပါ
**စိတ်ပိုင်းဆိုင်ရာ လူမှုရေးဆိုင်ရာထောက်ပံ့ပေးမှုအချက်အလက်များ**		
လူမှုရေးဆိုင်ရာထောက်ပံ့မှုရရှိခြင်း	☐ ရှိပါသည်	☐ မရှိပါ
စိတ်ပိုင်းဆိုင်ရာထောက်ပံ့မှုရရှိခြင်း	☐ ရှိပါသည်	☐ မရှိပါ

စိတ်ကျဆင်းမှုလက္ခဏာများအား ဆန်းစစ်ခြင်း

အောက်ပါအချက်များသည် သင်စိတ်ခံစားမှုများအား ကိုယ်စားပြုဖော်ပြပေးသည်။ ကျေးဇူးပြု၍ လွန်ခဲ့သောတစ်ပတ်အတွင်း အကြိမ်မည်မျှခံစားခဲ့ရသည်ကို ဖော်ပြပေးပါ။

	အကြောင်းအရာ	လွန်ခဲ့သော တစ်ပတ်အချိန်အတိုင်းအတာတွင်			
		ဖြစ်တောင့်ဖြစ်ခဲ (သို့) လုံးဝဖြစ်ခဲ (သို့) တစ်ရက်ထက်နည်း	အချို့အနည်းငယ်သောဖြစ်ခဲ (သို့) ၁-၂ ရက်	ရံဖန်ရံခါ (သို့) အချိန်အတော်များများ (သို့) ၃-၄ ရက်	အများအားဖြင့် (သို့) အချိန်တိုင်းနီးပါး (သို့) ၅-၇ ရက်
၁	ပုံမှန်အားဖြင့် ကျွန်ုပ်ကို စိတ်အနှောက်အယှက် မဖြစ်စေသော အရာများက စိတ်အနှောက်အယှက်ဖြစ်စေခဲ့သည်။.	☐	☐	☐	☐
၂	စားသောက်လိုစိတ်မရှိခဲ့ပါ။ အစားအသောက်ခံတွင်း ပျက်နေခဲ့သည်။	☐	☐	☐	☐
၃	ကျွန်ုပ်၏ မိသားစု (သို့) သူငယ်ချင်းမိတ်ဆွေများ၏ ကူညီဖေးမမှု ရှိသော်လည်း စိတ်နွမ်းလျ အားငယ်နေခြင်းကို မဖယ်ထုတ်နိုင်ဟု ခံစားခဲ့ရသည်။	☐	☐	☐	☐
၄	ကျွန်ုပ်သည် အခြားသူများနည်းတူ အဆင်ပြေနေသည်ဟု ခံစားမိခဲ့သည်။	☐	☐	☐	☐
၅	ကျွန်ုပ် ဆောင်ရွက်လုပ်ကိုင်နေသော အရာများအပေါ် စိတ်အာရုံမပြတ်ထားရှိရန် ခက်ခဲခဲ့သည်။	☐	☐	☐	☐
၆	ကျွန်ုပ် စိတ်ဓါတ်ကျနေခဲ့သည်။	☐	☐	☐	☐
၇	ကျွန်ုပ် ဆောင်ရွက်လုပ်ကိုင်ခဲ့သော အရာတိုင်းတွင် ကြိုးစားအားထုတ်မှုရှိခဲ့ရသည်။	☐	☐	☐	☐
၈	အနာဂတ်နှင့်ပတ်သတ်၍ မျှော်လင့်ချက်ရှိသည်ဟု ကျွန်ုပ် ခံစားခဲ့ရသည်။	☐	☐	☐	☐

၉	ကျွန်ုပ်၏ ဘဝသည် ကျရှုံးမှုတစ်ခုသာ ဖြစ်သည်ဟု တွေးမိခဲ့သည်။	☐	☐	☐	☐
၁၀	ကျွန်ုပ် စိုးရိမ်ကြောက်လန့်နေခဲ့သည်။	☐	☐	☐	☐
၁၁	ကောင်းမွန်နှစ်ခြိုက်စွာ အိပ်မရခဲ့ပါ။	☐	☐	☐	☐
၁၂	ကျွန်ုပ် ပျော်ရွှင်နေခဲ့သည်။	☐	☐	☐	☐
၁၃	ကျွန်ုပ် ပုံမှန်ထက် စကားပိုနည်းနေခဲ့သည်။	☐	☐	☐	☐
၁၄	အထီးကျန်ဆန်သည်ဟု ခံစားမိခဲ့သည်။	☐	☐	☐	☐
၁၅	လူများသည် ခင်မင်နှစ်လိုဖွယ် မကောင်းခဲ့ပါ။	☐	☐	☐	☐
၁၆	ဘဝတွင် နေပျော်ခဲ့သည်။	☐	☐	☐	☐
၁၇	ငိုကြွေးပူဆွေးရသည့် အချိန်များရှိခဲ့သည်။	☐	☐	☐	☐
၁၈	ကျွန်ုပ် ဝမ်းနည်းနေခဲ့ရသည်။	☐	☐	☐	☐
၁၉	လူအများက ကျွန်ုပ်ကို မနှစ်သက်ကြ ဟုခံစားမိခဲ့သည်။	☐	☐	☐	☐
၂၀	တစ်ခုခုကို စတင်လုပ်ကိုင်နိုင်စွမ်း မရှိခဲ့ပါ။	☐	☐	☐	☐
